# Supplementary material for: Unravelling the link between SARS-CoV-2 mutation frequencies, patient comorbidities, and structural dynamics
Source: PLoS One. 2024 Mar 14;19(3):e0291892. doi: 10.1371/journal.pone.0291892 (PMC10939192; doi:10.1371/journal.pone.0291892)
Supplement: S1 Table — (PDF) [file pone.0291892.s001.pdf]

S1 Table. Patient comorbidities including number of comorbidities, stage of COVID-19 infection and sequenced lineage of SARS-CoV-2 for each corresponding patient.

Comorbidities			COVID-19 severity		GISAID ID	Collection date	Age	Gender	Clade	Pango lineage
3 or more	2	1	Category 3, 4 and 5	Category 1 and 2						
No	No	No	No	No	EPI_ISL_5428560	02/06/2021	36	Male	GH	B.1.351
No	Yes	No	No	No	EPI_ISL_8745621	09/06/2021	51	Female	GK	B.1.617.2
Yes	No	No	No	No	EPI_ISL_6825270	09/06/2021	79	Female	GK	AY.51
Yes	No	No	No	No	EPI_ISL_5428562	09/06/2021	61	Female	GK	AY.51
Yes	No	Yes	No	No	EPI_ISL_5428561	09/06/2021	60	Female	GK	AY.51
Yes	No	Yes	No	Yes	EPI_ISL_4816832	09/06/2021	73	Female	GK	AY.51
Yes	No	No	No	No	EPI_ISL_7260006	10/06/2021	70	Female	GK	AY.51
No	No	No	No	No	EPI_ISL_6825272	10/06/2021	35	Female	GK	AY.51
Yes	No	No	No	No	EPI_ISL_6825271	10/06/2021	81	Female	GK	AY.51
Yes	No	No	No	No	EPI_ISL_6825263	10/06/2021	63	Female	GK	AY.51

Yes	No	No	No	Yes	EPI_ISL_4816833	10/06/2021	70	Female	GK	AY.53
No	No	No	No	No	EPI_ISL_8745614	17/06/2021	36	Female	GK	B.1.617.2
No	Yes	No	No	No	EPI_ISL_6825265	17/06/2021	28	Female	GK	AY.51
Yes	No	No	No	No	EPI_ISL_6825264	17/06/2021	68	Female	GK	AY.51
Yes	No	No	No	No	EPI_ISL_4071977	17/06/2021	59	Female	GK	AY.51
Yes	No	No	No	No	EPI_ISL_8745615	23/06/2021	83	Female	GK	B.1.617.2
No	No	No	No	Yes	EPI_ISL_6825266	26/06/2021	27	Male	GK	AY.59
No	No	Yes	No	No	EPI_ISL_8745616	30/06/2021	67	Female	GK	B.1.617.2
No	No	Yes	No	No	EPI_ISL_8745617	04/07/2021	74	Female	GK	AY.59
No	No	No	No	No	EPI_ISL_5742697	09/07/2021	37	Female	GK	AY.59
No	No	Yes	No	No	EPI_ISL_5742665	10/07/2021	36	Female	GK	AY.59
No	No	No	No	Yes	EPI_ISL_5742693	13/07/2021	35	Female	GK	AY.59
No	No	Yes	No	No	EPI_ISL_5742675	14/07/2021	41	Female	GK	AY.51
No	No	No	No	No	EPI_ISL_5742659	15/07/2021	43	Female	GK	AY.59
Yes	No	Yes	No	No	EPI_ISL_4071980	15/07/2021	48	Male	GK	AY.59

No	No	No	Yes	No	EPI_ISL_8745619	16/07/2021	36	Male	GK	B.1.617.2
No	No	No	No	No	EPI_ISL_5881287	17/07/2021	31	Female	GK	AY.51
Yes	No	No	No	No	EPI_ISL_4071978	18/07/2021	30	Female	GK	AY.59
Yes	No	No	No	No	EPI_ISL_4071979	19/07/2021	46	Female	GK	AY.59
No	No	No	No	Yes	EPI_ISL_5742682	21/07/2021	41	Male	GK	AY.59
No	Yes	No	No	No	EPI_ISL_4071983	30/07/2021	34	Male	GK	AY.59
Yes	No	No	Yes	No	EPI_ISL_4071981	30/07/2021	73	Female	GK	AY.59
No	No	No	Yes	No	EPI_ISL_4071984	01/08/2021	45	Male	GK	AY.59
No	No	No	No	Yes	EPI_ISL_3945546	02/08/2021	29	Female	GK	AY.59
Yes	No	No	Yes	No	EPI_ISL_3945545	02/08/2021	71	Female	GK	AY.59
Yes	No	No	No	No	EPI_ISL_3945548	09/08/2021	27	Male	GK	AY.59
No	No	No	Yes	No	EPI_ISL_3945547	09/08/2021	44	Male	GK	AY.59
No	No	Yes	No	No	EPI_ISL_4513510	16/08/2021	28	Female	GK	AY.59
No	Yes	No	No	No	EPI_ISL_4463195	16/08/2021	44	Female	GK	AY.59
No	No	No	No	Yes	EPI_ISL_4463194	16/08/2021	32	Female	GK	AY.79

No	No	No	No	No	EPI_ISL_4463193	16/08/2021	32	Female	GK	AY.59
No	No	No	No	No	EPI_ISL_4891935	10/09/2021	43	Female	GK	AY.59
No	No	No	No	No	EPI_ISL_4891929	13/09/2021	45	Female	GK	AY.59
No	Yes	No	No	No	EPI_ISL_4891941	19/09/2021	30	Female	GK	AY.59
No	No	No	No	Yes	EPI_ISL_4891939	19/09/2021	42	Female	GK	AY.59
No	Yes	No	No	No	EPI_ISL_4891938	19/09/2021	32	Female	GK	AY.59
No	No	No	No	No	EPI_ISL_4891942	20/09/2021	36	Female	GK	AY.59
No	No	No	No	No	EPI_ISL_4891946	22/09/2021	34	Female	GK	AY.59
Yes	No	No	No	No	EPI_ISL_4891940	22/09/2021	46	Male	GK	AY.59
Yes	No	No	Yes	No	EPI_ISL_5742681	02/10/2021	63	Female	GK	AY.59
No	No	No	No	Yes	EPI_ISL_5417593	04/10/2021	28	Female	GK	AY.24
No	No	No	No	No	EPI_ISL_5022688	04/10/2021	29	Female	GK	AY.59
No	No	No	No	No	EPI_ISL_5022682	04/10/2021	32	Female	GK	AY.59
No	No	No	No	Yes	EPI_ISL_5022675	04/10/2021	10	Male	GK	AY.59
Yes	No	No	No	No	EPI_ISL_5022673	04/10/2021	40	Female	GK	B.1.617.2

No	No	No	No	Yes	EPI_ISL_5022670	04/10/2021	47	Female	GK	AY.59
No	Yes	No	No	No	EPI_ISL_5428878	11/10/2021	38	Male	GK	AY.59
No	No	No	No	No	EPI_ISL_5428873	11/10/2021	41	Male	GK	AY.79
Yes	No	No	Yes	No	EPI_ISL_5428862	11/10/2021	59	Male	GK	AY.59
Yes	No	No	No	No	EPI_ISL_5428860	11/10/2021	71	Female	GK	AY.59
Yes	No	No	No	No	EPI_ISL_5417637	11/10/2021	31	Female	GK	AY.79
No	No	No	No	No	EPI_ISL_5417636	11/10/2021	unknown	Male	GK	AY.59
No	No	No	No	Yes	EPI_ISL_6452618	15/10/2021	2	Female	GK	AY.79
No	Yes	No	No	No	EPI_ISL_6452563	20/10/2021	38	Female	GK	AY.59
Yes	No	No	No	Yes	EPI_ISL_6452645	21/10/2021	34	Female	GK	AY.79
No	No	No	No	No	EPI_ISL_6452630	21/10/2021	25	Female	GK	AY.59
No	No	No	No	No	EPI_ISL_6452588	21/10/2021	39	Female	GK	AY.59
No	No	No	No	Yes	EPI_ISL_8408361	28/10/2021	3	Female	GK	AY.59
No	No	No	No	Yes	EPI_ISL_8408360	28/10/2021	7	Female	GK	AY.59
No	No	No	No	No	EPI_ISL_6452557	28/10/2021	37	Female	GK	AY.59

No	No	No	No	Yes	EPI_ISL_6567073	31/10/2021	29	Male	GK	AY.59
No	No	Yes	No	Yes	EPI_ISL_6567071	01/11/2021	24	Female	GK	AY.59
No	No	No	No	No	EPI_ISL_6567075	02/11/2021	24	Male	GK	AY.59
Yes	No	No	Yes	No	EPI_ISL_6567074	03/11/2021	36	Male	GK	AY.59
Yes	No	No	No	Yes	EPI_ISL_6567072	03/11/2021	35	Female	GK	AY.59
No	Yes	No	No	No	EPI_ISL_6567066	03/11/2021	5	Female	GK	AY.59
No	No	No	No	No	EPI_ISL_6705577	15/11/2021	34	Female	GK	AY.59
Yes	No	No	No	No	EPI_ISL_6705529	15/11/2021	58	Female	GK	AY.79
No	No	No	No	Yes	EPI_ISL_6705517	15/11/2021	34	Male	GK	AY.59
No	No	No	No	Yes	EPI_ISL_6705491	15/11/2021	35	Female	GK	AY.59
No	No	No	Yes	No	EPI_ISL_6567068	15/11/2021	80	Female	GK	AY.59
No	No	No	No	No	EPI_ISL_6567064	15/11/2021	26	Female	GK	AY.59
No	Yes	No	No	No	EPI_ISL_8438480	29/11/2021	31	Male	GK	AY.59
No	No	No	No	No	EPI_ISL_7464488	29/11/2021	36	Male	GK	B.1.617.2
No	No	No	No	Yes	EPI_ISL_7464487	29/11/2021	47	Male	GK	B.1.617.2

No	No	No	No	No	EPI_ISL_7464478	29/11/2021	38	Female	GK	AY.59
No	No	No	No	No	EPI_ISL_7464475	29/11/2021	20	Female	GK	B.1.617.2
No	No	No	No	Yes	EPI_ISL_7464472	29/11/2021	40	Female	GK	AY.59
No	No	No	No	No	EPI_ISL_7464471	29/11/2021	44	Male	GK	B.1.617.2
No	No	No	No	No	EPI_ISL_7464468	29/11/2021	52	Male	GK	AY.59
Yes	No	No	No	No	EPI_ISL_7831404	13/12/2021	66	Female	GK	AY.5
No	Yes	No	No	No	EPI_ISL_7831402	13/12/2021	41	Male	GK	AY.59
No	No	No	No	No	EPI_ISL_7831401	13/12/2021	48	Male	GK	AY.114
No	No	No	No	No	EPI_ISL_7831394	13/12/2021	11	Male	GK	AY.79
Yes	No	No	Yes	No	EPI_ISL_7831392	13/12/2021	50	Male	GK	B.1.617.2
No	No	No	No	No	EPI_ISL_7831390	13/12/2021	46	Male	GK	AY.79
Yes	No	No	Yes	No	EPI_ISL_7831387	13/12/2021	85	Male	GK	AY.59
No	No	Yes	No	No	EPI_ISL_7831385	13/12/2021	11	Male	GK	AY.59
No	No	No	No	No	EPI_ISL_8314874	28/12/2021	35	Male	GK	AY.79
