# Supplementary material for: Unravelling the link between SARS-CoV-2 mutation frequencies, patient comorbidities, and structural dynamics
Source: PLoS One. 2024 Mar 14;19(3):e0291892. doi: 10.1371/journal.pone.0291892 (PMC10939192; doi:10.1371/journal.pone.0291892)
Supplement: Attachment — Submitted filename: PONE-D-23-28307-RevMM.pdf [file pone.0291892.s002.pdf]

PLOS ONE

Unravelling the Link between SARS-CoV-2 Mutation Frequencies, Patient Comorbidities, and Structural Dynamics

--Manuscript Draft--

**Manuscript Number:**	PONE-D-23-28307
**Article Type:**	Research Article
**Full Title:**	Unravelling the Link between SARS-CoV-2 Mutation Frequencies, Patient Comorbidities, and Structural Dynamics
**Short Title:**	Linking structural mutations of SARS-CoV-2 to patient comorbidities and protein function
**Corresponding Author:**	Liyana Azmi<br>Universiti Sains Islam Malaysia<br>Nilai, MALAYSIA
**Keywords:**	SARS-CoV-2; comorbidities; mutations; Molecular dynamics
**Abstract:**	Genomic surveillance is crucial for tracking emergence and spread of novel variants of pathogens, such as SARS-CoV-2, to inform public health interventions and enforce control measures. However, in some settings where sequencing platforms are limited particularly in low- and middle-income countries, only certain patients can be selected for sequencing surveillance. Here, we show that patients with multiple comorbidities potentially harbour SARS-CoV-2 with higher mutation rates and thus deservedly require more attention for genomic surveillance. The relationship between the patient comorbidities, and type of amino acid mutations were assessed. Correlation analysis showed that there was a significant tendency for mutations to occur within the ORF1a region for patients with higher number of comorbidities. Frequency analysis of the amino acid substitution within ORF1a showed that nsp3 P822L of the PLpro protease was one of the highest occurring mutations. Using molecular dynamics, we simulated that the P822L mutation in PLpro represents a system with lower RMSD fluctuations, smaller Rg and RMSF - indicating a much stabler protein than the wildtype. The outcome of this study will help determine the relationship between the clinical status of a patient and the mutations of the infecting SARS-CoV-2 virus.
**Order of Authors:**	Liyana Azmi
	Amirah Azzeri
	Nurul Azmawati Mohamed
	Saarah Huurieyah Wan Rosli
	Muttaqillah Najihan Abdul Samat
	Zetti Zainol Rashid
	Muhammad Zarul Hanifah Md Zoqratt
	Muhammad Azamuddeen Mohammad Nasir
	Harpreet Kaur Ranjit Singh
	Muhamad Arif Mohamad Jamali
**Additional Information:**	
**Question**	**Response**
**Financial Disclosure**<br><br>Enter a financial disclosure statement that describes the sources of funding for the work included in this submission. Review the submission guidelines for detailed	"This study was funded by two the Universiti Sains Islam Malaysia Internal Research Grants: AA - Grant number PPPI/FPSK/0122/USIM/14622 and LA - Grant number PPPI/FPSK/0122/USIM/14322). The funders had no role in study design, data collection and analysis, decision to publish, or preparation of the manuscript."

Powered by Editorial Manager® and ProduXion Manager® from Aries Systems Corporation

requirements. View published research articles from *PLOS ONE* for specific examples.<br><br>This statement is required for submission and **will appear in the published article** if the submission is accepted. Please make sure it is accurate.<br><br>**Unfunded studies**<br>Enter: *The author(s) received no specific funding for this work.*<br><br>**Funded studies**<br>Enter a statement with the following details:<br>• Initials of the authors who received each award<br>• Grant numbers awarded to each author<br>• The full name of each funder<br>• URL of each funder website<br>• Did the sponsors or funders play any role in the study design, data collection and analysis, decision to publish, or preparation of the manuscript?<br>• **NO** - Include this sentence at the end of your statement: *The funders had no role in study design, data collection and analysis, decision to publish, or preparation of the manuscript.*<br>• **YES** - Specify the role(s) played.<br><br>* typeset	
**Competing Interests**<br><br>Use the instructions below to enter a competing interest statement for this submission. On behalf of all authors, disclose any competing interests that could be perceived to bias this work—acknowledging all financial support and any other relevant financial or non-financial competing interests.<br><br>This statement is required for submission and **will appear in the published article** if the submission is accepted. Please make sure it is accurate and that any funding sources listed in your Funding Information later in the submission form are also declared in your Financial Disclosure	The authors have declared that no competing interests exist.

statement.<br><br>View published research articles from *PLOS ONE* for specific examples.<br><br>**NO authors have competing interests**<br><br>Enter: *The authors have declared that no competing interests exist.*<br><br>**Authors with competing interests**<br><br>Enter competing interest details beginning with this statement:<br><br>*I have read the journal's policy and the authors of this manuscript have the following competing interests: [insert competing interests here]*<br><br>* typeset	
**Ethics Statement**<br><br>Enter an ethics statement for this submission. This statement is required if the study involved:<br><br>• Human participants<br>• Human specimens or tissue<br>• Vertebrate animals or cephalopods<br>• Vertebrate embryos or tissues<br>• Field research<br><br>Write "N/A" if the submission does not require an ethics statement.<br><br>General guidance is provided below. Consult the submission guidelines for detailed instructions. **Make sure that all information entered here is included in the Methods section of the manuscript.**	Ethics approval for the study was granted by UKMMC Research Ethics Committee (JEP-2022-805).

**Format for specific study types**<br><br>**Human Subject Research (involving human participants and/or tissue)**<br>• Give the name of the institutional review board or ethics committee that approved the study<br>• Include the approval number and/or a statement indicating approval of this research<br>• Indicate the form of consent obtained (written/oral) or the reason that consent was not obtained (e.g. the data were analyzed anonymously)<br><br>**Animal Research (involving vertebrate animals, embryos or tissues)**<br>• Provide the name of the Institutional Animal Care and Use Committee (IACUC) or other relevant ethics board that reviewed the study protocol, and indicate whether they approved this research or granted a formal waiver of ethical approval<br>• Include an approval number if one was obtained<br>• If the study involved *non-human primates*, add *additional details* about animal welfare and steps taken to ameliorate suffering<br>• If anesthesia, euthanasia, or any kind of animal sacrifice is part of the study, include briefly which substances and/or methods were applied<br><br>**Field Research**<br><br>Include the following details if this study involves the collection of plant, animal, or other materials from a natural setting:<br>• Field permit number<br>• Name of the institution or relevant body that granted permission	
**Data Availability**<br><br>Authors are required to make all data underlying the findings described fully available, without restriction, and from the time of publication. PLOS allows rare exceptions to address legal and ethical concerns. See the PLOS Data Policy and FAQ for detailed information.	Yes - all data are fully available without restriction

A Data Availability Statement describing where the data can be found is required at submission. Your answers to this question constitute the Data Availability Statement and **will be published in the article**, if accepted.<br><br>**Important:** Stating 'data available on request from the author' is not sufficient. If your data are only available upon request, select 'No' for the first question and explain your exceptional situation in the text box.<br><br>Do the authors confirm that all data underlying the findings described in their manuscript are fully available without restriction?	
**Describe where the data may be found in full sentences. If you are copying our sample text, replace any instances of XXX with the appropriate details.**<br><br>• If the data are **held or will be held in a public repository**, include URLs, accession numbers or DOIs. If this information will only be available after acceptance, indicate this by ticking the box below. For example: *All XXX files are available from the XXX database (accession number(s) XXX, XXX.*).<br>• If the data are all contained **within the manuscript and/or Supporting Information files**, enter the following: *All relevant data are within the manuscript and its Supporting Information files.*<br>• If neither of these applies but you are able to provide **details of access elsewhere**, with or without limitations, please do so. For example:<br><br>*Data cannot be shared publicly because of [XXX]. Data are available from the XXX Institutional Data Access / Ethics Committee (contact via XXX) for researchers who meet the criteria for access to confidential data.*<br><br>*The data underlying the results presented in the study are available from (include the name of the third party*	All sequences used in this files are available from the GISAID database. The comorbidity status for each patient are available within the Supporting Information files.

and contact information or URL).<br>• This text is appropriate if the data are owned by a third party and authors do not have permission to share the data.<br><br>* typeset	
Additional data availability information:	Tick here if the URLs/accession numbers/DOIs will be available only after acceptance of the manuscript for publication so that we can ensure their inclusion before publication.

Manuscript
Click here to access/download;Manuscript;Manuscript_LA_edited.docx

Abstract

Genomic surveillance is crucial for tracking emergence and spread of novel variants of pathogens, such as SARS-CoV-2, to inform public health interventions and enforce control measures. However, in some settings where sequencing platforms are limited particularly in low- and middle-income countries, only certain patients can be selected for sequencing surveillance. Here, we show that patients with multiple comorbidities potentially harbour SARS-CoV-2 with higher mutation rates and thus deservedly require more attention for genomic surveillance. The relationship between the patient comorbidities, and type of amino acid mutations were assessed. Correlation analysis showed that there was a significant tendency for mutations to occur within the ORF1a region for patients with higher number of comorbidities. Frequency analysis of the amino acid substitution within ORF1a showed that nsp3 P822L of the PLpro protease was one of the highest occurring mutations. Using molecular dynamics, we simulated that the P822L mutation in PLpro represents a system with lower RMSD fluctuations, smaller Rg and RMSF - indicating a much stabler protein than the wildtype. The outcome of this study will help determine the relationship between the clinical status of a patient and the mutations of the infecting SARS-CoV-2 virus.

Introduction

The coronavirus disease-2019 (COVID-19) was first isolated from Wuhan City, Hubei Province, China in December 2019. It is caused by Severe Acute Respiratory Syndrome Coronavirus 2 (SARS-CoV-2,) a novel betacoronavirus (β-CoV). The SARS-CoV-2 genome showed 79% homology to SARS-CoV therefore possesses similarities of its pathogenesis, epidemiology, viral origin, and mechanism of action to SARS-CoV (1). Symptoms of SARS-

CoV-2 infection vary widely in humans, ranging from mild flu-like symptoms such as fever, fatigue, and dry cough to complete respiratory failure (2). Similar to severe acute respiratory syndrome (SARS), transmission of COVID-19 is airborne, fomites, and air droplets (3). The highly contagious nature of the virus prompted widespread lockdowns on a global scale to curb its spread.

In Malaysia, the first wave of COVID-19 infection with a total of 22 cases was first recorded on January 25th, 2020. Up until August 2023, there were a total of 5,121,858 COVID-19 cases with 37,165 deaths (COVID-19 | KKMNOW, 2023.). Incidence for COVID-19 was highest amongst the age group of 55-64 and 63% of those >60 years were reported to be fatal. In addition, 81% of those above 60 years had chronic comorbidities such as diabetes, and heart disease (4). Recent studies have shown that persistent and immunocompromised patients are more likely to harbour SARS-CoV-2 viruses with enhanced mutation rates (5,6) .

Coronaviruses, including SARS-CoV-2, exhibit significant genetic diversity and mutate rapidly. Amino acid substitution within the viral structural proteins including S (spike), E (envelope), M (membrane), N (nucleocapsid), accessory proteins, and ORF1a/ORF1ab polyproteins are capable of altering traits like pathogenicity and transmissibility, which also produces more virulent and infectious variants of SARS-CoV-2 (7,8). The emergence of more virulent and transmissible variants of SARS-CoV-2 have caused a surge in the number of cases and death tolls, collapsing many health systems. To address these challenges, frequent genomic surveillance is essential to track the emergence of new variants and their impacts. Shared virus sequences had been published in the Global Initiative on Sharing All Influenza Data (GISAID) database (9) to enable real-time genomic surveillance on a global scale (10). Up until August

2023, approximately 15 million complete and partial genomes of SARS-CoV-2 are deposited in GISAID. However, the number from Malaysia is notably lower compared to other countries, potentially hindering the ability to detect and respond to emerging variants.

Considering this context, this study describes the associations between patient comorbidities with SARS-CoV-2 mutation types and numbers. We also analysed the mutation rates for SARS-CoV-2, particularly those residing within the ORF1a region. Finally, we investigated selected mutations of high frequencies and demonstrated via molecular dynamics, how the mutation potentially contributes to increased transmissibility of COVID-19.

Methodology

Sampling and ethics approval

Data analysis was conducted on retrospective 99 clinical histories of patients infected with COVID-19 from Hospital Canselor Tuanku Muhriz Universiti Kebangsaan Malaysia (HCTM) dating from 2nd June 2021 to 28th December 2021. The corresponding SARS-CoV-2 sequences of the infected patients were extracted from GISAID on the 4th of May 2022 and the retrospective clinical histories of the patients were collected from the 26th January 2023 until 11th April 2023. Each sequence deposition was matched to the patient's clinical history which states the type of comorbidities diagnosed at the time of sampling for COVID-19. All the patient’s information was made fully anonymous prior to any analysis. Ethics approval for the study was granted by the Research Ethics Committee, HCTM (JEP-2022-805).

Mutation analysis

COVID-19 metadata was obtained from GISAID database and narrowed between the period of 1st June 2021 until 31st December 2021. Global AY.59 genomes and metadata was obtained from the GISAID database (11) between the period of 2nd June 2021 until 28th December 202; this is accessible with EPI_SET_230816ue (10.55876/gis8.230816ue). Visualisation of the data analysis is done using seaborn and matplotlib Python packages (12,13)

A phylogenetic tree of the global AY.59 was constructed based on the Nextstrain SARS-CoV-2 workflow using software IQ-TREE and Augur version 22.3.0 (14–16). The phylogenetic tree was time-scaled using TreeTime (17). Phylogenetic tree was visualised using baltic Python package (https://github.com/blab/baltic).

Statistical analysis

Statistical analysis was performed using SPSS version 24.0 (SPSS Inc., Chicago, Illinois, USA). Results of descriptive analysis of sociodemographic characteristics, clinical characteristics, and the outcomes were presented for all patients and by disease stages. Visual assessment and normality tests, such as the Kolmogorov-Smirnov test, were used to test for normality of distribution prior to conducting and reporting the results of the descriptive analysis. Where appropriate, continuous variables were presented as means and standard deviations (SD). For findings that were not normally distributed, median and inter-quartile range (IQR) was used. For categorical variables, results were presented as frequencies and percentages. The association between quantitative independent variable such as the number of comorbidities, and dependent variables were determined using Pearson's (Spearman's for non-

normally distributed data) correlation test. Results for the bivariate analysis were presented with p-value and the correlation coefficient when appropriate.

Molecular dynamics

The nsp3 protease 3D structure was extracted from the protein databank (PDB) with the PDB ID: 7D7K. mutation for the residue P822L (in the structure was identified as residue 77) was performed using the mutagenesis function and visualised using PyMOL (The PyMOL Molecular Graphics System, Version 2.0 Schrödinger, LLC). Each system consisted of the protein-protein complex in a solvated dodecahedron box with a minimum distance of 1.2 nm from the boundary. The systems were filled with single-point charge water and subsequently neutralized by adding counter cations (Na^+) or anions (Cl^-) (18). The solvated systems were then energy minimized using the steepest descent method (19), followed by the equilibrium for 1000 ps through NVT and NPT ensembles to optimize the orientation and system density. The final equilibrated systems were used as starting conformations to run the MD simulations for 1000 ns. Finally, the output trajectories were obtained, and the estimation of Root Mean Square Deviation (RMSD), Root Mean Square Fluctuation (RMSF), Solvent Accessible Surface Area (SASA), and Radius of Gyration (Rg) was done using GROMACS packages. The graphs were analysed using XMGRACE software.

Results

Demography and correlation analysis

In this study, a total of 99 sequences were extracted from GISAID and matched to the patient clinical data dating from 2nd June 2021 to 28th December 2022. Among these sequences, 67 patients (67%) were from female individuals and 32 (32%) from male individuals, with the majority falling within the age group of 30-39 years old (Fig 1A). Sorting the 99 SARS-CoV-2 genomes (S1Table) by pangolin lineage revealed a notable dominance of AY.59 (61%), followed by AY.51 (13%), B.1.617.2 (11%), AY.79 (9%), and AY.42, B.1.351, AY.5, AY.114, and AY.53, each representing 1% (Fig 1B). We constructed a phylogenetic tree to then observe if there is a dominance of the AY.59 lineage in other countries (Fig 1C). Based on our phylogenetics, the AY.59 was shown to dominate Malaysia, circulate mostly within the Southeast Asian regions and was only minimally detected in other parts of the globe.

Fig. 1 Patient demography and phylogenetic distribution of SARS-CoV-2 from 2nd June 2021 to 28th December 2022. (A) Age and sex distribution of our samples showed that the largest age group is 30-39 years old, with most samples being females (n=67, 67%). (B) 61.6% of the circulating Pango lineage is represented by AY.59. (C) Phylogenetic tree representing distribution of AY.59 in Malaysia, Indonesia, Thailand, Singapore, and other countries.

SARS-CoV-2 mutation analysis correlating with patient comorbidities.

The patients included in this study were assorted based on the number of comorbidities diagnosed and the severity of their COVID-19 infections at the time of sample collection for SARS-CoV-2 sequencing. The type of comorbidities noted are those which are known to cause a reduction of immune status of a patient and includes the following: chronic heart, kidney,

liver and lung diseases, diabetes melitus, dyslipidemia, inflammatory and infectious diseases including HIV/AIDS. The number of comorbidities were classified according to clinical diagnoses, and the severity categories were recorded during the sampling period (Table 1). Among the categorised patients, the highest proportions were those with no known medical illnesses (32%), followed by patients with three or more comorbidities (30%), two comorbidities (11%), and a single comorbidity (10%). The severities of the patients were sorted based on COVID-19 categories with the following category 1 (asymptomatic) & 2 (symptomatic with no pneumonia), category 3 (symptomatic with pneumonia), 4 (symptomatic with pneumonia requiring supplemental oxygen) and 5 (critically ill with or without other organ failures) (20).

Table 1. Distribution of patient comorbidities and severities.

**Groups**	**Total (n=99)**
**Comorbidity**	
3 and above comorbidities	27 (27%)
2 comorbidities	10 (10%)
1 comorbidity	16 (16%)
No known medical illness	46 (46%)
**COVID-19 severity**	
Category 1 & 2	80 (80%)
Category 3, 4 & 5	19 (19%)

To determine the potential associations between SARS-CoV-2 mutations, patient comorbidities, and severities, we utilised a correlation analysis accordingly. We investigated the number of highest occurring mutations within the SARS-CoV-2 genome and sorted the mutations based on their respective genes/regions. The genomic distribution of amino acid substitutions revealed that the ORF1a gene had the highest number of amino acid substitutions (n = 92), followed by ORF1b (n = 51), S (n = 42), N (n = 20), M (n = 3), and finally E (n = 2) (Table 2).

Table 2. Genomic distribution of the identified SARS-CoV-2 mutations.

**Gene/region**	***n* of amino acid substitutions**
ORF1a	92
ORF1b	51
S	42
N	20
M	3
E	2

Since ORF1a showed the highest number of occurring mutations, we performed correlation analysis between the number of ORF1a mutations The results indicated a significant link between the number of ORF1a mutations and number of comorbidities (p value < 0.05). There were moderate-positive correlations between the number of comorbidities and number of ORF1a mutations, with higher number of comorbidities associated with higher number of

mutations [$r=0.05$, $p<0.05$]. We also tested the correlation between COVID-19 severities with the number of ORF1a mutations. However, possibly, due to limited available data on the severity status of patients, our study found insignificant correlations between the number of mutations and the severity of COVID-19 infections.

ORF1a mutation profiles

The ORF1a gene contains essential non-structural proteins crucial for the viral replication machinery and the maintenance of the viral genome. Adaptive mutations occurring in ORF1a/b might enhance viral replication, drug resistance, and increased virulence. We also questioned if mutations within this region could lead to the enhanced ability of SARS-CoV-2 to infect a patient with comorbidities. Consequently, since ORF1a displayed the highest number of mutations, we further analysed the mutation locations within the gene and sorted them based on their frequencies for each patient (Fig 2). Mutation analysis of the ORF1a region revealed up to 92 amino acid (AA) substitutions in our samples. Sorting of the mutation frequencies revealed the top 5 mutations occurred to be nsp6 V149A, nsp4 A446V, nsp3 P822L, nsp6 T181I and nsp2 P129L (Table 3).

Fig 2. Frequency of mutations in ORF1a. The Y-axis lists the number of patient samples and X-axis lists the frequency and type of mutations per patient.

Table 3: The top five occurring mutations within ORF1a.

Gene	AA substitution	No. of occurrence (n)	Frequencies (%)
nsp6	V149A	82/99	82.8
nsp4	A446V	82/99	82.8
nsp3	P822L	81/99	81.8
nsp6	T181I	63/99	63.6
nsp2	P129L	19/99	19.1

Since the mutation nsp3 P822L (hereon referred to as P822L) encodes for the papain-like protease (PLpro), we were interested to know if there was a correlation between this mutation and patient comorbidities, severity as well as infectivity. We were interested to know if P822L correlated with patient comorbidities. Do patients harbouring SARS-CoV-2 with P822L cause enhanced COVID-19 severity and are more transmissible? We performed a correlational analysis of P822L with patients with comorbidity status. Our results indicate trends that suggest higher comorbidities and severe COVID-19 for patients harbouring P822L. However, our results were insignificant due to the small sample size.

Molecular dynamic simulation of P822L

Notably, the PLpro plays a crucial role in facilitating viral replication by cleaving the ORF1ab polyprotein into functional segments (nsp1-nsp3). Additionally, it serves as a mechanism for the virus to evade the host's immune response by eliminating the interferon-

stimulating gene-15 protein (ISG15) from host proteins, thus disrupting the host's innate antiviral defense. Previous studies suggest that mutations in PLpro can lead to alterations in the enzyme's specificity, activity, and causing reductions in antiviral effectiveness (21).

The potential impact of the P822L mutation on PLpro stability was tested using molecular dynamics (MD) simulations on the apo structure of the SARS-CoV-2 PLPro protease (PDB ID: 7D7K). Using PyMOL, we generated a model containing the mutant P822L (Fig 3). To assess protein stability, MD simulations of the wild-type (WT) and P822L were performed over a 1000 ns timescale. Parameters including RMSD, RMSF, and Rg were employed for analysis.

Fig 3. Generation of P822L of PLpro. The (A) apo form of PLpro was extracted from PDB ID: 7D7K. The P822L was generated and visualised using PyMOL. Here, we present a (B) zoomed view of WT residue P822 and (C) mutated L822.

The RMSD analysis is a measure of protein stability. The RMSD plot (Fig 4A) indicates that P822L displayed significantly more stable mechanics after approximately 550 ns. At this point, P822L reached equilibrium, whereas the WT continued to exhibit fluctuations post 550 ns. Notably, the dip in stability observed at 1000 ns for the WT suggests its ongoing instability. The reduced fluctuation implies that the mutation might have reduced the protein's flexibility, potentially enhancing its resistance to environmental changes. Overall, the average RMSD values throughout the entire simulation times for WT (0.341 nm) were also higher compared to P822L (0.302 nm).

The RMSF of the complex were analysed to assess the flexibility and rigidity of bound complexes. RMSF involves observing the C-alpha atom of the models' residues to infer each atom's fluctuations across the C-alpha backbone. Based on the RMSF simulation (Fig 4B), the change of P822 to L822 showed fluctuation, as observed in residue 77. Additionally, two other areas (residues K254 and S294) showed significantly higher fluctuations in the WT compared with the mutant.

We also analysed Rg values to assess the compactness and rigidity of both proteins. The WT and P822L exhibited initial fluctuations during the first 200 ns, with P822L stabilising from 500 ns onwards. Compared with the WT structure, the mutated version displayed notably stable values throughout the simulation, suggesting a potentially more stable system (Fig 4C). The better stabilities for the mutated structure suggest that the P822L mutation might have primarily impacted the protein's structure at a localised level.

Fig 4. Molecular dynamic simulations of WT and P822L. The (A) RMSD, (B) RMSF simulation with a closed-up view on residues 200-345 and (C) Rg simulations of WT (blue) and P822L (red) indicates that the P822L is a stabler protein compared with the WT.

Discussion

Our genomic analysis of 99 samples revealed a prominent prevalence of the Pango AY.59 lineage during Malaysia's third wave of COVID-19. Interestingly, another study noted

a dominance of AY.79 on the West coast of Malaysia, indicating the infection patterns of the infected patients during June – December 2021 (22). Notably, the Delta variant is notable for its heightened virulence and prompted us to explore the correlation between patient comorbidity levels and the manifestation of SARS-CoV-2 mutations.

Various factors contribute to COVID-19 transmission, including patient demographics like sex, age, and ethnicity, which may impact disease outcomes. In this study, we investigated the number of patient comorbidities that could play a role in manifesting SARS-CoV-2 mutations. Are patients with more comorbidities more favourable hosts for viral replication, leading to beneficial mutations for viral transmissibility? Our study has revealed a significant link between higher comorbidity counts and enhanced mutations within the SARS-CoV-2 virus. Previous studies show that comorbidities associated with higher ACE2 expression may enhance the virus entry and the severity of COVID-19 infection (23). It is possible that comorbidities contribute to the enhanced systemic inflammation, release of reactive oxygen species which drives mutations occur due to the biochemical process occurring inside the living organism such as errors in replication, editing, or damage to a nucleic acid (24). Notably, the rate of viral mutations also depends on community characteristics like sex, age (25), ethnicities (26), and host genetic variabilities (27). Furthermore, the different types of selective pressures determine the types of mutations to occur. Wilkinson et al., (2022) showed that in long-term infections, there is a tendency to partly select for mutations which aid the virus with *intra*-host replication (cell-to-cell transmission) and persistence as opposed to the general SARS-CoV-2 population, where mutations, which aid *inter*-host transmission are more strongly selected. Work by Maurya et al. (2022) supported our findings, as they identified infrequent mutations

in recovered COVID-19 patients, implying the existence of localised mutation pools and a lack of preferential selection by the virus.

Regarding COVID-19 severity, when we performed a correlational analysis to explore the potential link between COVID-19 severity and the number of patient comorbidities, we found the connection between these factors to be statistically insignificant. Notably, other studies have shown that patient immune status may have a role in diversifying mutations in SARS-CoV-2. For example, a case report from Hensley et al., (2021) showed prolonged SARS-CoV-2 infection in a patient with multiple myeloma. Extraction and genomic profiling of the virus demonstrated high viral replication and diversification within the patient prior to the patient’s death. Potentially, future studies could demonstrate the relation of COVID-19 severities with SARS-CoV-2 mutations by adopting a larger sample size. We also noted that our study only considered the number of comorbidities and did not consider the type of comorbidities with the number and type of SARS-CoV-2 mutations. Particularly, hypertension, diabetes mellitus, and coronary artery diseases have been shown to contribute to disease severity and susceptibility for in patients to be infected by SARS-CoV-2 (31). Future work can then delve into the type of comorbidities and associate this with the type of mutations within SARS-CoV-2.

The increase in undiagnosed COVID-19 mutations in people with comorbidities poses a serious public health risk. Comorbidities compromise the immune system and predisposes a person to more severe illness when they contract the virus. When combined with highly transmissible variants such as Delta and Omicron, the impact can be particularly severe, leading to higher rates of infection in the population (32). This could lead to increased demand on health systems, which could be overwhelmed by an increase in severe cases, putting pressure

on medical resources and health professionals. In addition, people with comorbidities who were infected with a variant of concern were more likely to require hospitalisation and intensive care (33). This highlights the importance of vigilant surveillance and detection of variants, especially in populations with pre-existing health conditions.

The presence of undetected variations in people with comorbidities may affect the effectiveness of vaccination. Variants with mutations that allow them to partially bypass immunity, such as Omicron, may reduce the protective benefits of vaccination and previous infections. This increases the risk of severe disease in both vaccinated and unvaccinated patients with concomitant disease. A multi-pronged strategy is needed to reduce the burden of undetected variation in patients with comorbidities. This includes increased genomic surveillance to detect new variations and investigate their potential impact on disease severity and vaccine efficacy. In addition, targeted public health interventions (34), such as promoting vaccination campaigns specifically aimed at people with comorbidities can help reduce the overall burden of disease within this vulnerable group.

Following SARS-CoV-2 transmissibility, we investigated the second highest number of mutations within the samples, which is the P822L of the PLpro. The PLpro domain of nsp3 is a highly conserved domain, which encodes for host cell survival signalling pathways (35) and thus implies lower mutation rates within this region compared to other viral regions such as the spike protein. However, it was interesting to note that for our tested samples, the PLpro showed high mutation frequencies of nsp3 P822L. Abbasian et al., (2023) reported high mutation rates (over 50%) in the nsp3 region. On the other hand, Anwar et al., (2022) described P822L to be a single occurring mutation of their tested samples. Interestingly, the recurrent

mutations of P822L were also identified in immunodeficient patients (28), supporting the notion that a host's genetic and or disease status do play a role in fostering beneficial mutations for SARS-CoV-2.

We questioned if the P822L might play a role in attenuating or increasing viral fitness or infectivity. Throughout the evolution of the virus there were various mutations in the same site aside from ORF1a P1640L, specifically ORF1a P1640S (36), and ORF1a P1640L. This suggests that these mutations on this site led to the stability of the protein folding could confer enhanced virulence of SARS-CoV-2. Naderi et al., (2023) showed that nsp3 protease localises to the deubiquitinating site in the PLpro domain which overlaps with the ISG15 binding site, suggesting it may modulate host antiviral responses. Indeed, in our study, we show that the P822L is a stabilising mutation, and suggest a protein with improved function by suppressing the impact of other deleterious mutations (39). We tracked the P822L mutation across lineages and found that P822L from the Delta lineage is retained and continues to occur in the Omicron lineage (40). Furthermore, our RMSD data, indicating fluctuations post 550 ns, highlights the importance of extended simulations in other systems. Future in vitro experiments can provide valuable insights into the role of P822L towards nsp3 stability.

Conclusion

Genetic surveillance is a crucial tool for scouring variants and analysing infection patterns within populations. Furthermore, the availability of an open-sourced database with genomic information enabled us to assess the role of patient status such as comorbidities in COVID-19 infections with SARS-CoV-2 mutations. Future studies should include larger

sample sizes to assess the role of SARS-CoV-2 mutations with COVID-19 severity. Notably, the type of SARS-CoV-2 mutation also, could potentially affect the type and number of patient comorbidities, and severity of the COVID-19 infections. We showed trends suggesting a possible association between P822L and higher comorbidity rates and severe COVID-19 outcomes but was limited due to the small sample size. Notably, the nsp3 P822L mutation of the PLpro domain exhibited one of the highest mutation frequencies, suggesting its potential role in viral replication enhancement and virulence.

Our molecular dynamics simulations indicated that P822L exhibited increased stability compared to the wild-type structure, potentially resulting in enhanced resistance to environmental fluctuations. Fluctuation analysis further revealed that P822L might have led to reduced flexibility in certain regions of the protease, potentially affecting its function. Overall, our findings underscore the need for more extensive research and larger datasets to elucidate the intricate connections between SARS-CoV-2 mutations, patient characteristics, and disease outcomes.

In summary, our study provides valuable insights into the demography and genetic diversity of SARS-CoV-2 genomes, sheds light on potential correlations between mutations and comorbidities, and introduces intriguing implications of the nsp3 P822L mutation through molecular dynamics simulations. While our findings hold promising avenues for further investigation, their significance awaits confirmation through larger-scale studies and enhanced data availability.

Acknowledgements

We also gratefully acknowledge all SARS-CoV-2 data contributors, i.e., the Authors, their originating and submitting laboratories responsible for obtaining the specimens, including Mohd Noor bin Mat Isa and Nor Azila Muhammad Azami for generating the genetic sequence and metadata and sharing via the GISAID Initiative, on which this research is based. Ethics approval by Research Ethics Committee, HCTM (JEP-2022-805).

References

1. Lu H, Stratton CW, Tang Y. Outbreak of pneumonia of unknown etiology in Wuhan, China: The mystery and the miracle. J Med Virol. 2020;92(4):401–2. Available from: https://onlinelibrary.wiley.com/doi/abs/10.1002/jmv.25678

2. Tian J, Yuan X, Xiao J, Zhong Q, Yang C, Liu B, et al. Clinical characteristics and risk factors associated with COVID-19 disease severity in patients with cancer in Wuhan, China: a multicentre, retrospective, cohort study. Lancet Oncol. 2020;21(7):893–903. Available from: https://pubmed.ncbi.nlm.nih.gov/32479790/

3. Liu J, Liao X, Qian S, Yuan J, Wang F, Liu Y, et al. Community Transmission of Severe Acute Respiratory Syndrome Coronavirus 2, Shenzhen, China, 2020. Emerg Infect Dis. 2020;26(6):1320–3. Available from: https://pubmed.ncbi.nlm.nih.gov/32125269/

4. Hashim MJ, Alsuwaidi AR, Khan G. Population risk factors for COVID-19 mortality in 93 countries. J Epidemiol Glob Health. 2020 Sep 1;10(3):204–8.

5. Corey L, Beyrer C, Cohen MS, Michael NL, Bedford T, Rolland M. SARS-CoV-2 Variants in Patients with Immunosuppression. N Engl J Med. 2021;385(6):562–6. Available from: https://pubmed.ncbi.nlm.nih.gov/34347959/

6. Kemp SA, Collier DA, Datir RP, Ferreira IATM, Gayed S, Jahun A, et al. SARS-CoV-2 evolution during treatment of chronic infection. Nature. 2021;592(7853):277–82. Available from: https://pubmed.ncbi.nlm.nih.gov/33545711/

7. Harvey WT, Carabelli AM, Jackson B, Gupta RK, Thomson EC, Harrison EM, et al. SARS-CoV-2 variants, spike mutations and immune escape. Nat Rev Microbiol. 2021;19(7):409–24. Available from: https://pubmed.ncbi.nlm.nih.gov/34075212/

8. Yan R, Zhang Y, Li Y, Xia L, Guo Y, Zhou Q. Structural basis for the recognition of SARS-CoV-2 by full-length human ACE2. Science. 2020;367(6485):1444–8. Available from: http://science.sciencemag.org/

9. Gralinski LE, Menachery VD. Return of the Coronavirus: 2019-nCoV. Viruses 2020, Vol 12, Page 135. 2020;12(2):135. Available from: https://www.mdpi.com/1999-4915/12/2/135/htm

10. Meredith LW, Hamilton WL, Warne B, Houldcroft CJ, Hosmillo M, Jahun AS, et al. Rapid implementation of SARS-CoV-2 sequencing to investigate cases of healthcare associated COVID-19: a prospective genomic surveillance study. Lancet Infect Dis. 2020;20(11):1263–72. Available from: https://pubmed.ncbi.nlm.nih.gov/32679081/

11. Khare S, Gurry C, Freitas L, Schultz MB, Bach G, Diallo A, et al. GISAID’s Role in Pandemic Response. China CDC Wkly. 2021;3(49):1049–51. Available from: https://pubmed.ncbi.nlm.nih.gov/34934514/

12. Hunter JD. Matplotlib: A 2D graphics environment. Comput Sci Eng. 2007;9(3):90–5.

13. Waskom M, Botvinnik O, O’Kane D, Hobson P, Lukauskas S, Gemperline DC, et al. mwaskom/seaborn: v0.8.1. 2017; Available from: https://zenodo.org/record/883859

14. Minh BQ, Schmidt HA, Chernomor O, Schrempf D, Woodhams MD, Von Haeseler A, et al. IQ-TREE 2: New Models and Efficient Methods for Phylogenetic Inference in the Genomic Era. Mol Biol Evol. 2020;37(5):1530. Available from: /pmc/articles/PMC7182206/

15. Hadfield J, Megill C, Bell SM, Huddleston J, Potter B, Callender C, et al. Nextstrain: real-time tracking of pathogen evolution. Bioinformatics. 2018;34(23):4121–3. Available from: https://pubmed.ncbi.nlm.nih.gov/29790939/

16. Huddleston J, Hadfield J, Sibley T, Lee J, Fay K, Ilcisin M, et al. Augur: a bioinformatics toolkit for phylogenetic analyses of human pathogens. J Open Source Softw. 2021;6(57):2906. Available from: https://pubmed.ncbi.nlm.nih.gov/34189396/

17. Sagulenko P, Puller V, Neher RA. TreeTime: Maximum likelihood phylodynamic analysis. Virus Evol. 2018;4(1). Available from: https://pubmed.ncbi.nlm.nih.gov/29340210/

18. Gorb L, Hill FC, Kholod Y, Muratov EN, Kuz’min VE, Leszczynski J, et al. Progress in Predictions of Environmentally Important Physicochemical Properties of Energetic Materials: Applications of Quantum-Chemical Calculations. Practical Aspects of Computational Chemistry II. 2012;335–59. Available from: https://link.springer.com/chapter/10.1007/978-94-007-0923-2_9

19. Petrova SS, Solov’Ev AD. The Origin of the Method of Steepest Descent. Historia Mathematica. 1997 Nov 1;24(4):361–75.

20. MOH. Clinical Management of Confirmed COVID-19 in Adult and Paediatric. 2022; Available from: https://www.cdc.gov/coronavirus/2019-ncov/science/science-

21. Alabbas AB, Alamri MA. Analyzing the effect of mutations in SARS-CoV2 papain-like protease from Saudi isolates on protein structure and drug-protein binding: Molecular modelling and dynamics studies. Saudi J Biol Sci. 2022;29(1):526. Available from: /pmc/articles/PMC8447498/

22. Muhammad Azami NA, Perera D, Thayan R, Bakar SA, Sam IC, Salleh MZ, et al. SARS-CoV-2 genomic surveillance in Malaysia: displacement of B.1.617.2 with AY lineages as the dominant Delta variants and the introduction of Omicron during the fourth epidemic wave. Int J Infect Dis. 2022 Dec;125. Available from: https://pubmed.ncbi.nlm.nih.gov/36336246/

23. Chatterjee S, Nalla LV, Sharma M, Sharma N, Singh AA, Malim FM, et al. Association of COVID-19 with Comorbidities: An Update. ACS Pharmacol Transl Sci. 2023;6(3):334–54. Available from: https://pubs.acs.org/doi/full/10.1021/acsptsci.2c00181

24. Khan MZI, Nazli A, Al-furas H, Asad MI, Ajmal I, Khan D, et al. An overview of viral mutagenesis and the impact on pathogenesis of SARS-CoV-2 variants. Front Immunol. 2022 Nov 28; 13:1034444.

25. Saha O, Islam I, Shatadru RN, Rakhi NN, Hossain MS, Rahaman MM. Temporal landscape of mutational frequencies in SARS-CoV-2 genomes of Bangladesh: possible implications from the ongoing outbreak in Bangladesh. Virus Genes. 2021;57(5):413. Available from: /pmc/articles/PMC8274265/

26. Vadgama N, Kreymerman A, Campbell J, Shamardina O, Brugger C, Research Consortium GE, et al. SARS-CoV-2 Susceptibility and ACE2 Gene Variations Within Diverse Ethnic Backgrounds. Front Genet. 2022; 13:1. Available from: /pmc/articles/PMC9091502/

27. Ovsyannikova IG, Haralambieva IH, Crooke SN, Poland GA, Kennedy RB. The role of host genetics in the immune response to SARS- CoV- 2 and COVID- 19 susceptibility and severity. Immunol Rev. 2020;296(1):205. Available from: /pmc/articles/PMC7404857/

28. Wilkinson SAJ, Sparks N, Kele B, Peacock TP, Robson SC, Connor TR, et al. Recurrent SARS-CoV-2 mutations in immunodeficient patients. Virus Evol. 2022;8(2). Available from: https://dx.doi.org/10.1093/ve/veac050

29. Maurya R, Mishra P, Swaminathan A, Ravi V, Saifi S, Kanakan A, et al. SARS-CoV-2 Mutations and COVID-19 Clinical Outcome: Mutation Global Frequency Dynamics and Structural Modulation Hold the Key. Front Cell Infect Microbiol. 2022 Mar 21; 12:868414.

30. Hensley MK, Bain WG, Jacobs J, Nambulli S, Parikh U, Cillo A, et al. Intractable Coronavirus Disease 2019 (COVID-19) and Prolonged Severe Acute Respiratory Syndrome Coronavirus 2 (SARS-CoV-2) Replication in a Chimeric Antigen Receptor-Modified T-Cell Therapy Recipient: A Case Study. Clinical Infectious Diseases. 2021 ;73(3): e815–21. Available from: https://academic.oup.com/cid/article/73/3/e815/6122591

31. Feng S, Song F, Guo W, Tan J, Zhang X, Qiao F, et al. Potential Genes Associated with COVID-19 and Comorbidity. Int J Med Sci. 2022;19(2):402. Available from: /pmc/articles/PMC8795808/

32. Willett BJ, Grove J, MacLean OA, Wilkie C, De Lorenzo G, Furnon W, et al. SARS-CoV-2 Omicron is an immune escape variant with an altered cell entry pathway. Nature Microbiology 2022 7:8. 2022;7(8):1161–79. Available from: https://www.nature.com/articles/s41564-022-01143-7

33. Veneti L, Seppälä E, Storm ML, Salamanca BV, Buanes EA, Aasand N, et al. Increased risk of hospitalisation and intensive care admission associated with reported cases of SARS-CoV-2 variants B.1.1.7 and B.1.351 in Norway, December 2020 –May 2021. PLoS One. 2021;16(10). Available from: /pmc/articles/PMC8504717/

34. Scendoni R, Fedeli P, Cingolani M. The Network of Services for COVID-19 Vaccination in Persons with Mental Disorders: The Italian Social Health System, Its Organization, and Bioethical Issues. Front Public Health. 2022; 10:870386. Available from: /pmc/articles/PMC9252269/

35. Osipiuk J, Azizi SA, Dvorkin S, Endres M, Jedrzejczak R, Jones KA, et al. Structure of papain-like protease from SARS-CoV-2 and its complexes with non-covalent inhibitors. Nat Commun. 2021;12(1). Available from: /pmc/articles/PMC7854729/

36. Abbasian MH, Mahmanzar M, Rahimian K, Mahdavi B, Tokhanbigli S, Moradi B, et al. Global landscape of SARS-CoV-2 mutations and conserved regions. J Transl Med. 2023 Dec 1;21(1):152

37. Anwar MZ, Lodhi MS, Sharif S, Khan MT, Khan MI. Coronavirus Genomes and Unique Mutations in Structural and Non-Structural Proteins in Pakistani SARS-CoV-2 Delta Variants during the Fourth Wave of the Pandemic. Genes (Basel). 2022 Mar 1, 13(3):552. Available from: https://www.mdpi.com/2073-4425/13/3/552/htm

38. Naderi S, Chen PE, Murall CL, Poujol R, Kraemer S, Pickering BS, et al. Zooanthroponotic transmission of SARS-CoV-2 and host-specific viral mutations revealed by genome-wide phylogenetic analysis. Elife. 2023 Apr 1;12.

39. Zimmerman MI, Hart KM, Sibbald CA, Frederick TE, Jimah JR, Knoverek CR, et al. Prediction of New Stabilizing Mutations Based on Mechanistic Insights from Markov State Models. ACS Cent Sci. 2017;3(12):1311–21. Available from: https://pubs.acs.org/doi/full/10.1021/acscentsci.7b00465

40. Saifi S, Ravi V, Sharma S, Swaminathan A, Chauhan NS, Pandey R. SARS-CoV-2 VOCs, Mutational diversity and clinical outcome: Are they modulating drug efficacy by altered binding strength? Genomics. 2022 Sep 1;114(5):110466.

Supporting Information

S1 Table. Patient comorbidities including number of comorbidities, stage of COVID-19 infection and sequenced lineage of SARS-CoV-2 for each corresponding patient.

Figure 1

Click here to access/download;Figure;Fig1.tif

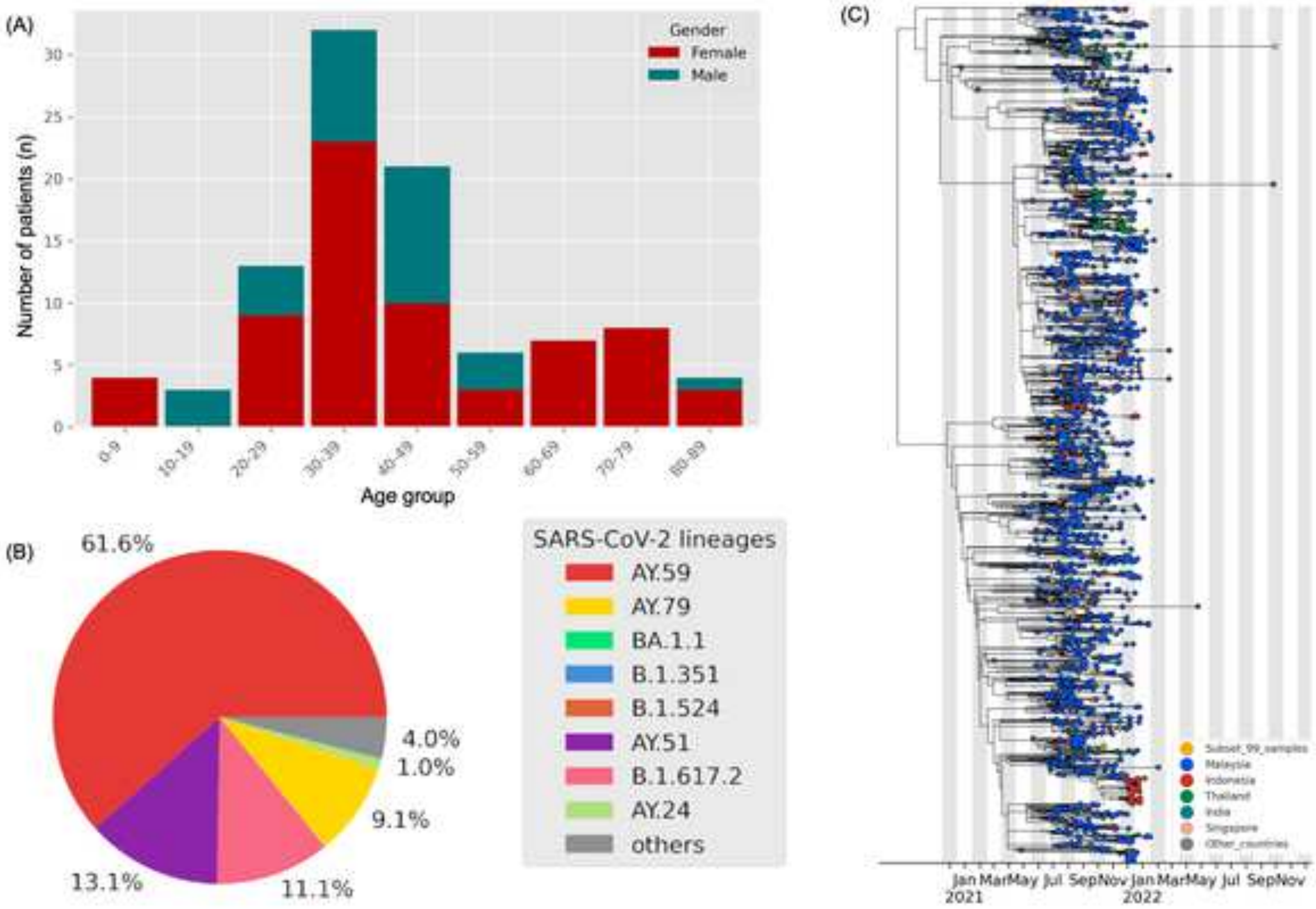

Figure 2

Click here to access/download;Figure;Fig2.tif

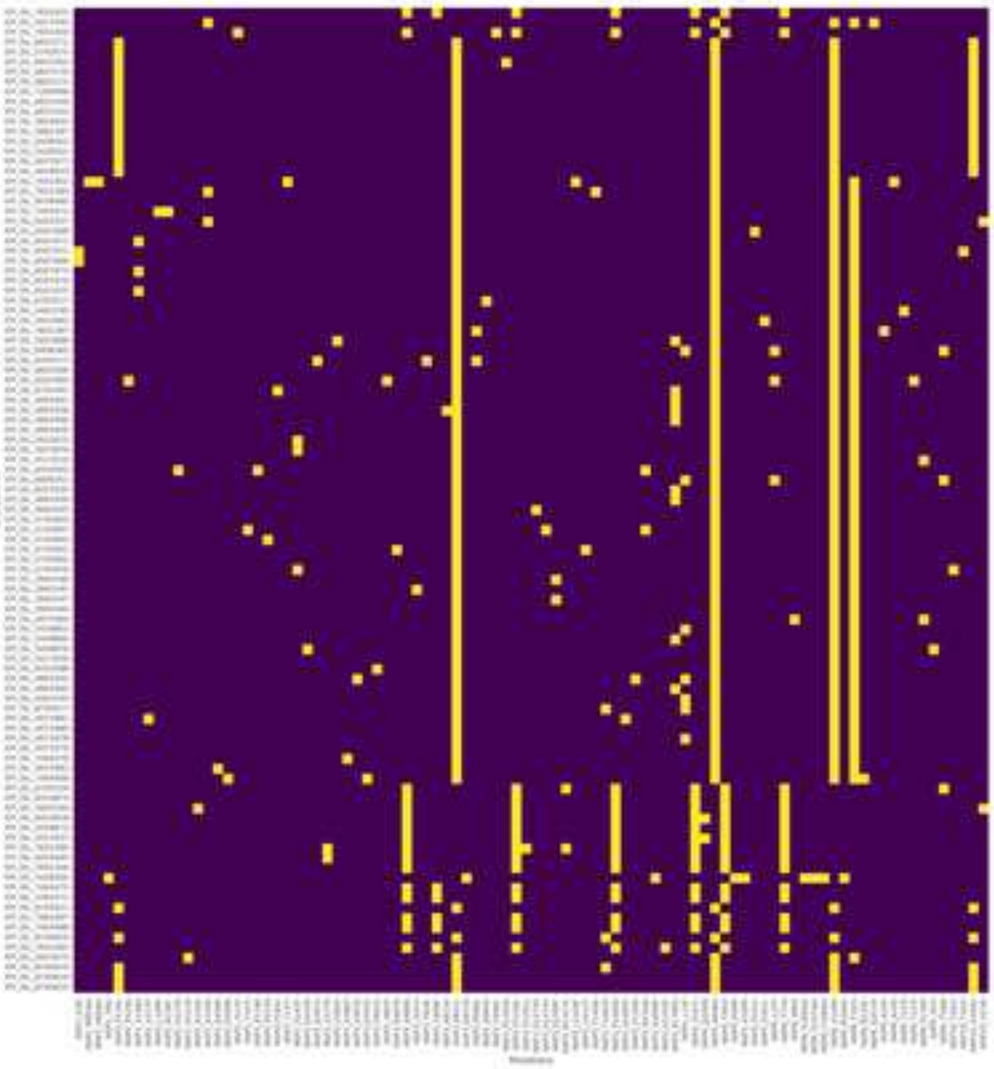

 Click here to access/download;Figure;Fig3.tif

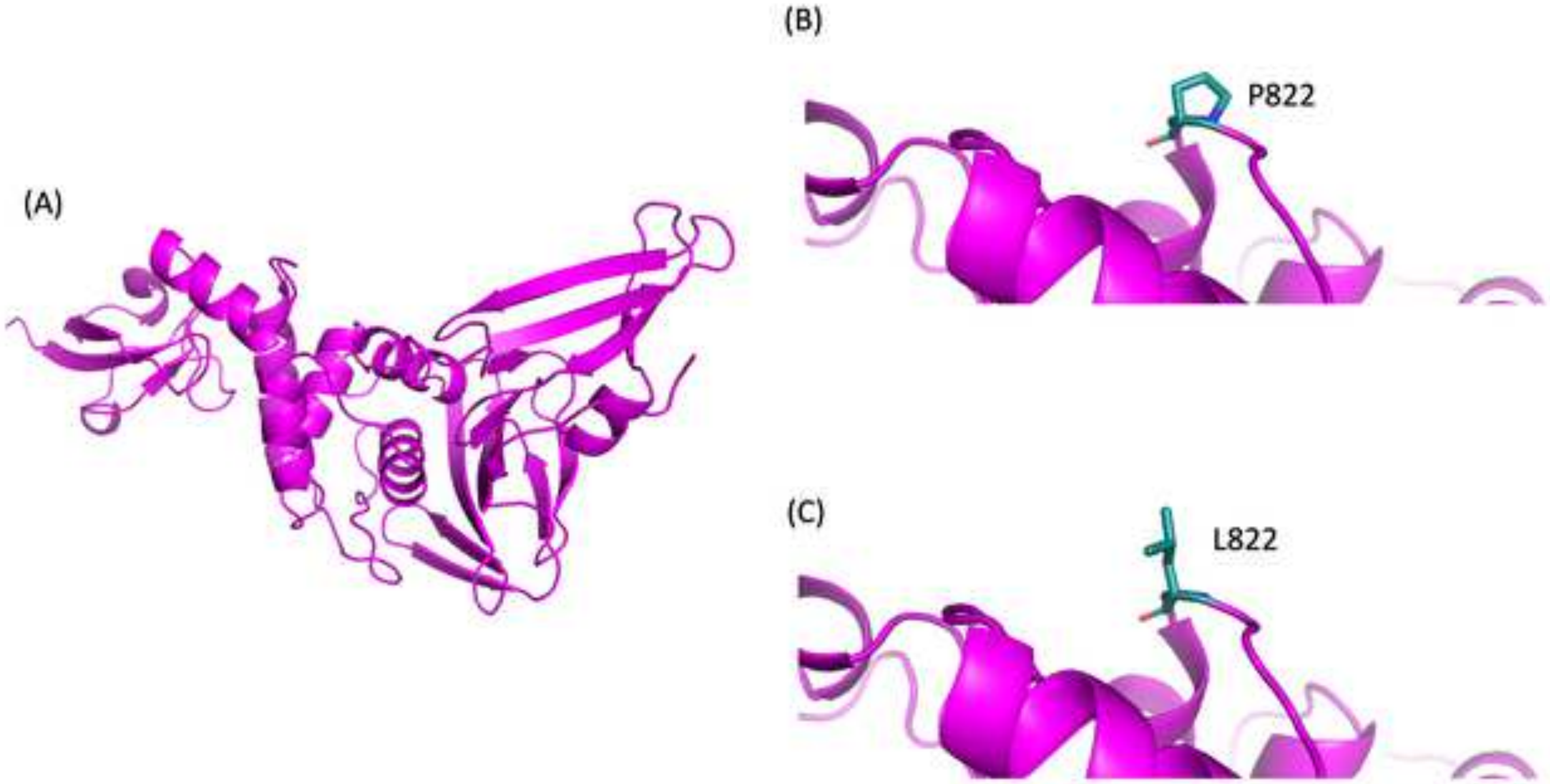

Figure 4

Click here to access/download;Figure;Fig4.tif

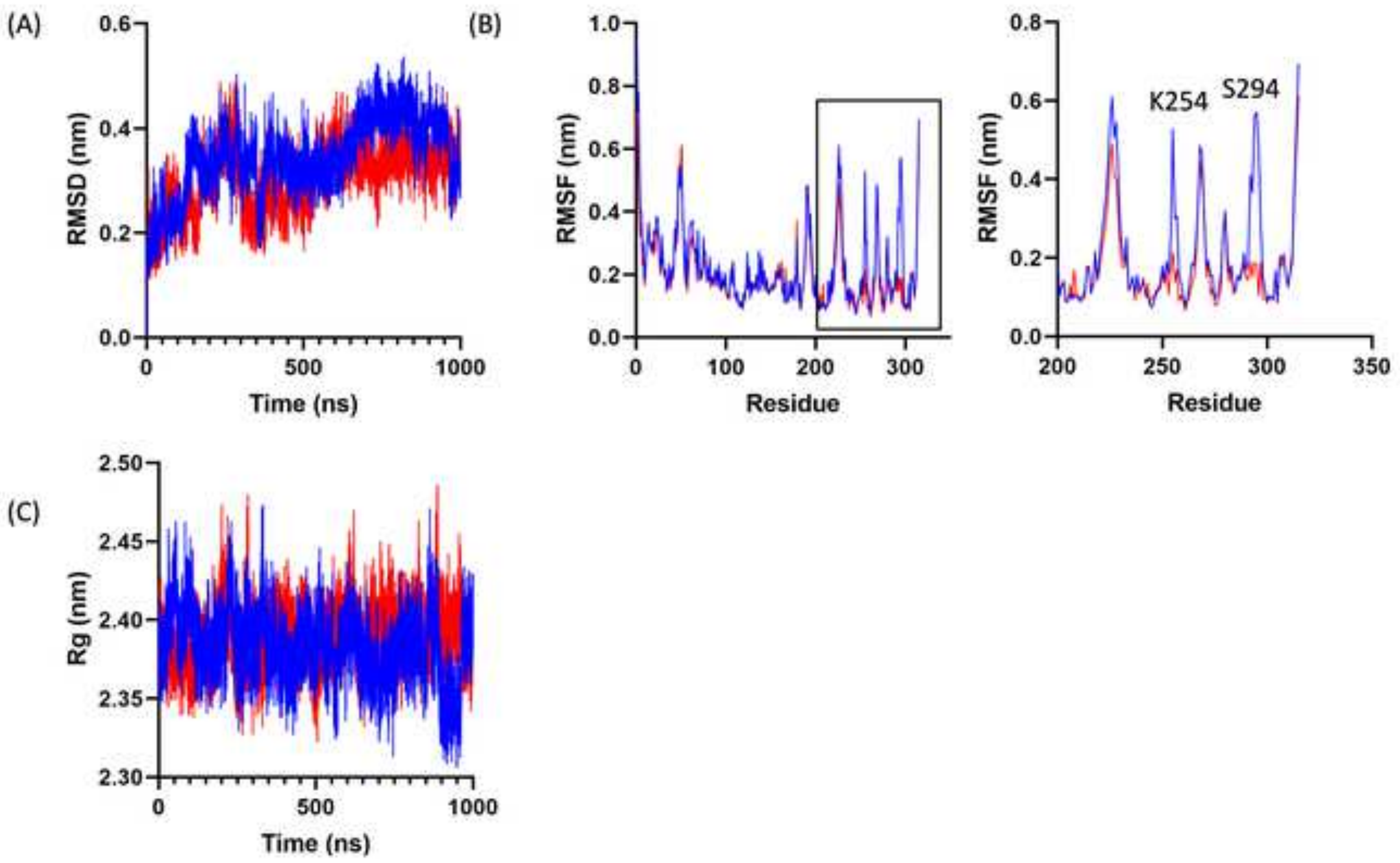

S1 Table

Click here to access/download
Supporting Information
Supporting information.pdf
