# Supplementary material for: Unravelling the link between SARS-CoV-2 mutation frequencies, patient comorbidities, and structural dynamics
Source: PLoS One. 2024 Mar 14;19(3):e0291892. doi: 10.1371/journal.pone.0291892 (PMC10939192; doi:10.1371/journal.pone.0291892)
Supplement: Attachment — Submitted filename: Response to Reviewers.docx [file pone.0291892.s003.docx]

Liyana Azmi
Faculty of Medicine and Health Sciences, Universiti Sains Islam Malaysia, Bandar Baru Nilai, 71800 Nilai, Negeri Sembilan, Malaysia

31^st^ August 2023

Dear Editor,

We would like to thank the reviewers in evaluating our manuscript titled “*Unravelling the Link between SARS-CoV-2 Mutation Frequencies, Patient Comorbidities, and Structural Dynamics,"* submitted under Manuscript ID PONE-D-23-28307 to PLOS ONE. Your insightful comments has been helpful in refining our manuscript.

In this rebuttal letter, I would like to highlight the key points raised by the reviewers and outline the specific changes made to address each comment.

| Reviewer | Comment | Response |
| --- | --- | --- |
| 1 | The authors adopted the PDB.file (7D7K) for performing the computational analysis which is deposited in its monomeric state. Typically, the PLPro is a homodimer relating to its biological activity (https://doi.org/10.1021/acsinfecdis.1c00631) and so computational study is better within its homodimeric state since it is the active form of the protein. Authors should provide a rational for adopting monomer state for computational analysis. | It was an oversight from our side that we performed our computational analysis of the PLPro in monomer state. To that end, we would like to replace the monomer results with the homodimer form.  In the original manuscript, we show that the mutated form is more stable compared with the WT. When we performed the homodimer analysis, this state was much more amplified and adds to the significance of our results. To this end, we have presented our new results and discussions in the updated manuscript.  Additionally, we have added a rational for choosing the homodimer form of PLPro in the following sentence:  “However, seeing that the active form of PLpro is a homodimer relating to its biological activity, we chose to perform the simulations on the bat SARS-CoV PLpro homodimer, BtSCoV-Rf1.2004 (PDB ID: 7SKQ). Furthermore, PLPro from BtSCoV-Rf1.2004 shares high sequence similarity with PLpro from SARS-CoV-2 (82%) (22) and warrants our basis for selecting the homodimer form of PLpro for analysis”  We would like to once again, thank the reviewer for this insightful comment. |
|  | Authors should provide a rational for performing computational analysis on an adopted apo over holo state of the PLpro protein. | We chose to adopt the apo state of PLPro over its holo state to elaborate its conformational changes prior to binding to its ligands.   Our results show that the WT form exhibits high RMSD and reflects conformational changes, which might not be visible should we opt for its holo form. |
|  | Authors are advised to better schematically represent Table 2 highlighting the location of the gene regions. | We agree with the reviewer that Table 2 alone would not represent our data best. To this end, we have added a lollipop plot (Fig 2) to support Table 2, which also maps out the frequencies and location of the amino acid substitutions. The figure was highlighted in this sentence :  “We investigated the number of highest occurring mutations within the SARS-CoV-2 genome and mapped the frequency of mutations based on their respective genes/regions (Fig 2).” |
|  | Authors announced their monitoring for the molecular dynamics SASA trajectories; however, this figure is missing. It would be interesting to visualize such data. | We apologise for the oversight on our part, and have included and discussed the significance of the SASA trajectories for both systems.   We express our gratitude to the reviewers for their thorough analysis of this section. |
|  | In Figure 3A, it is better to highlight the mutated residue first, prior zooming in | We have amended the figure and highlighted the mutated residue prior zooming in. |
|  | All dates need to be revised thoroughly, either those of this study at their different occurrences in the manuscript and of other cited work. | We thank the reviewer for the detailed assessment of our dates in this study. To this end, we have edited several sections to better deliver our data, particularly in the methods section:  “The corresponding SARS-CoV-2 sequences of the infected patients were extracted from GISAID and the retrospective clinical histories of the patients matching to the SARS-CoV-2 sequences were collected.”  We have also corrected other inconsistent dates within this manuscript. |
| 2 | The manuscript is well written in terms of the language and clarity of statements. However, some words need to be revised and modified for further clarity, notably:  a. Line 34 -> “persistent”.  b. Line 151 -> “Genomic distribution of amino acid substitutions ...”.  c. Line 168 -> “contains”.  d. Line 257 -> “occur”.  e. Line 353 -> “demography”. | We thank the reviewer for these comments and have refined the specified terms to enhance the clarity of our sentences as the following:   a. Line 34 -> “persistent”.   Revised to: chronic diseases  b. Line 151 -> “Genomic distribution of amino acid substitutions ...”.  Revised to “frequencies of amino acid substitutions”  c. Line 168 -> “contains”.  Revised to “encodes”  d. Line 257 -> “occur”.  Revised by omitting “occur”. The new sentence is now as following: ‘It is possible that comorbidities contribute to the enhanced systemic inflammation releases reactive oxygen species and drives mutations. Changes in the the biochemical process have been shown to induce errors in replication, editing, or damage to a nucleic acid (24).’  e. Line 353 -> “demography”.  Revised by omitting “demography” |
|  | In line 103, There are unresolved abbreviations that need to be elucidated. The abstract should have the full wording of any abbreviations as it is mostly read separately. | We have added full terms for all abbreviations where necessary. Similarly for the abstract, we have included full wordings for the provided abbreviations. |
|  | In line 139 in the Results section, the percentages summarized in text from Table 1 were different than those in the table, so please revise. | We have corrected the numbers in the table to match the text. |
|  | In Figure 1 and more than once in the text, Age and sex were included as important factors in the rate of viral mutation (as in line 114 in the Results and line 259 in the Discussion). However, the significance of these factors was not addressed in the study which was carried out using a dataset with a 2:1 female to male ratio. The authors need to show how sex could impact their results | We thank the reviewer for this insightful comment. To address this, we have include the following input in our discussion:  “Regarding the sex ratio of our dataset, we utilised a convenience sampling approach, thus demonstrating an unbiased scenario of COVID-19 patients of HCTM within the specified time. However, our dataset featured a 2:1 female-to-male ratio. We performed a correlation analysis between gender and the frequency of amino acid substitutions. However, there was no statistically significant association between gender and the number of mutations, although males reported higher mutations than females [p=0.769]. Based on this trend, it is likely that males are predisposed towards generating higher frequencies of SARS-CoV-2 mutations. However, a bigger sample size is warranted to fully comprehend the significance of sex with mutation frequencies for SARS-CoV-2.” |
|  | In line 265 in the Discussion section, the statement “work of ...... supported our findings”, is somehow confusing as the reader here may need to know which findings in particular are consistent with the cited reference. | We apologise for the misleading sentence in this section and have reword the line to the following:  “Work by Maurya et al. (2022) supported our findings, as they identified a single mutation (S194L) to frequently occur in their mortality group, implying the exclusivity or tendency for mutations to occur in patients with severe disease progressions.” |

In addition to the above comments, all spelling and grammatical errors pointed out by the reviewers have been corrected.

We look forward to hearing from you in due time regarding our submission and to respond to any further questions and comments you may have.

Sincerely,

Liyana Azmi
