# Supplementary material for: Unravelling the link between SARS-CoV-2 mutation frequencies, patient comorbidities, and structural dynamics
Source: PLoS One. 2024 Mar 14;19(3):e0291892. doi: 10.1371/journal.pone.0291892 (PMC10939192; doi:10.1371/journal.pone.0291892)
Supplement: Attachment — Submitted filename: Response to Reviewers_2.docx [file pone.0291892.s005.docx]

Liyana Azmi
Faculty of Medicine and Health Sciences, Universiti Sains Islam Malaysia, Bandar Baru Nilai, 71800 Nilai, Negeri Sembilan, Malaysia

15^th^ February 2024

Dear Editor,

We would like to thank the reviewers in evaluating our manuscript titled “*Unravelling the Link between SARS-CoV-2 Mutation Frequencies, Patient Comorbidities, and Structural Dynamics,"* submitted under Manuscript ID PONE-D-23-28307 to PLOS ONE. Your insightful comments has been helpful in refining our manuscript.

In this rebuttal letter, I would like to highlight the key points raised by the reviewers and outline the specific changes made to address each comment.

| Reviewer | Comment | Response |
| --- | --- | --- |
| 2 | In line 251, WT and the mutant were mixed up. Which one has the lower average RMSD value?. | We thank the reviewer for the comment, and acknowledge that this was an error form our part.  We have fixed this information to include the correct RMSD to the following sentence:  ‘The average RMSD values throughout the entire simulation times for P822L (1.68 nm) was lower compared to WT (2.86 nm). Additionally, since the WT possesses a higher maximum RMSD, the energy landscape and conformational changes within the WT is more significant compared with P822L.’ |
|  | In line 371, I highlighted this in the last round, but no response received. I will be clearer. Does the phrase mean that aside from ORF1a P1640L, specifically P1640S, and P1640L again? Isn't the former P1460L the exact latter P1460L? | We thank the reviewer for the comment and apologise for missing this note during the first correction. This was again, an oversight from our end and only meant to mention P1640S.  We also fixed the sentence structure to the following: ‘Throughout the virus's evolution, various mutations in the same residue occurred, one of them being ORF1a P1640S (37).’ |
|  | A thorough check of spelling and punctuations are still needed for a best shape.  I again uploaded a highlighted copy of the manuscript for pointing at my comments. | We have sought assistance from an English-proof reader to correct the grammatical and sentence structure errors within this manuscript. |

We look forward to hearing from you in due time regarding our submission and to respond to any further questions and comments you may have.

Sincerely,

Liyana Azmi
